# Micropore closure time is longer following microneedle application to skin of color

**DOI:** 10.1038/s41598-020-75246-8

**Published:** 2020-11-03

**Authors:** Abayomi T. Ogunjimi, Jamie Carr, Christine Lawson, Nkanyezi Ferguson, Nicole K. Brogden

**Affiliations:** 1 Division of Pharmaceutics and Translational Therapeutics, Department of Pharmaceutical Sciences and Experimental Therapeutics, University of Iowa College of Pharmacy, 180 South Grand Avenue, 552 CPB, Iowa City, IA 52242-1112 USA; 2 Department of Dermatology, Carver College of Medicine, University of Iowa, Iowa City, IA USA

**Keywords:** Drug delivery, Health care, Medical research

## Abstract

Microneedles (MNs) allow transdermal delivery of skin-impermeable drugs by creating transient epidermal micropores, and micropore lifetime directly affects drug diffusion timeframes. Healthy subjects (n = 111) completed the study, self-identifying as Asian (n = 32), Bi-/multi-racial (n = 10), Black (n = 22), White (n = 23), Latino (n = 23), and Native American/Hawaiian (n = 1). L* was measured with tristimulus colorimetry to objectively describe skin lightness/darkness. MNs were applied to the upper arm; impedance and transepidermal water loss (TEWL) were measured at baseline and post-MN to confirm micropore formation. Impedance was repeated for 4 days to determine micropore lifetime. Post-MN changes in TEWL and impedance were significant in all groups (*p* < 0.05), confirming micropore formation regardless of skin type. Micropore lifetime was significantly longer in Blacks (66.5 ± 19.5 h) versus Asians (44.1 ± 14.0 h), Bi-/multi-racial (48.0 ± 16.0 h), and Whites (50.2 ± 2.6 h). Latinos (61.1 ± 16.1 h) had significantly longer micropore closure time versus Asians (44.1 ± 14.0 h). When categorizing data according to L*, micropore lifetime was significantly longer in darker skin. We report for the first time that micropore lifetime differences are present in human subjects of different ethnic/racial backgrounds, with longer micropore lifetime in skin of color. These results also suggest that objectively measured skin color is a better predictor of micropore lifetime than self-identified race/ethnicity.

## Introduction

Application of drugs to the skin for systemic delivery comes with many advantages, including consistent plasma concentrations, avoidance of first-pass hepatic metabolism, and ease of use by patients. The success of transdermal delivery is highly dependent upon the ability of a drug to cross the stratum corneum, the outermost epidermal layer that imparts a barrier to the absorption of chemicals or the loss of water. Differences in barrier and structural skin properties have been reported amongst human subjects of different ethnic/racial backgrounds, including stratum corneum cohesion and strength, transepidermal water loss (TEWL), skin elasticity, epidermal and dermal thickness, and ceramide content^1–4^. The effect of these properties on the extent and variability of dermal absorption remains under debate with conflicting results reported in the literature, and this is an area in need of more study.

For drugs that cannot effectively cross the stratum corneum, microneedles (MNs) offer an opportunity for transdermal delivery of a broad range of therapeutics including hydrophilic small molecular weight drugs, peptides, proteins, and vaccines^5,6^. MNs are micron-scale projections that when applied to the skin create aqueous epidermal channels known as micropores which effectively bypass the stratum corneum. MNs can be used as single needles or in arrays of hundreds of MNs, in a variety of materials including silicone, ceramics, metals, polymers, sugars, and hydrogels. MNs offer advantages such as faster onset of action and improved bioavailability over conventional topical delivery methods, and better patient compliance, less pain, and the possibility of self-administration when compared to intravenous injections^7–9^. The uses of MNs have also expanded from basic delivery of small or large molecules to biosensing, where body fluid analytes such as glucose, biomarkers, alcohol, and drug concentrations can be measured continuously and pain-free. This is significant in that MNs used for biosensing could eliminate the need for blood extraction by hypodermic needles and consequently reduce associated problems such as infection, sample contamination, and challenges with obtaining venous access (i.e., in pediatric and geriatric patients)^10–12^. MNs can be used in a pretreatment approach in which an array of solid MNs is used to create micropores in the skin, followed by application of a drug patch or semisolid formulation to the treated skin area. The active drug ingredient enters into the micropores and then diffuses into the lower skin layers. This method has been well studied and shown to have promising clinical benefits^13,14^.

The success of a MN pretreatment approach is critically dependent on the continuous availability of open micropores. Innate functional and structural skin characteristics, such as those seen between differing skin types, may influence the time to micropore closure after MN application. The idea that differing skin properties may affect micropore closure timeframes is supported by previous studies that demonstrated slower micropore closure times in elderly vs. young healthy subjects, which could be a result of the decreased elasticity and stratum corneum hydration typically found in aging skin^15^. Understanding micropore closure kinetics in different racial/ethnic skin types may help to identify specific delivery windows for transdermal drug delivery in various population subgroups. Tailored drug formulations and application schedules may be necessary for successful therapeutic outcomes with MN-assisted drug delivery in diverse patient populations, and this could have significant implications in guiding product development strategies.

To date there have been no formal studies comparing micropore closure kinetics in human subjects of different skin types. It is also unknown if differences in epidermal skin characteristics influence restoration of epidermal barrier integrity after MN application. Here we present the first study demonstrating that there are significant differences in micropore closure timeframes after MN application in a diverse population of healthy human subjects.

## Results and discussion

In this study we aimed to investigate how ethnic/racial differences can influence cutaneous micropore closure after MN application. Administration of drugs and vaccines with MN-assisted delivery has gained traction in recent years with several successes in the delivery of various therapeutics in vitro, in vivo in animals and particularly, in human clinical studies^13,14,16,17^. Some benefits offered by MN-assisted delivery of both drugs and vaccines may be especially well suited for developing countries around the world, which will include large populations of people from diverse ethnic/racial backgrounds. The demographics in the United States are also rapidly changing and increasing in diversity. Thus, it is clear that emerging MN therapeutics need to be suitable and safe for broad populations of patients, which makes it critical that we understand how different ethnic/racial skin types respond to MN treatment.

Delivering drugs through the skin with the MN pretreatment approach depends on multiple variables, including the physical characteristics of the MNs and physicochemical properties of the drug molecule to be delivered. These variables can be controlled for during the study design phase. What cannot be easily controlled for is the lifetime of the micropores that are created by MN insertion. Closure of the micropores depends on innate skin barrier function characteristics that have been shown to differ in people from diverse ethnic/racial backgrounds^15,18^. Understanding these differences and how they affect micropore lifetime will give us insight into the window that is available for optimum drug delivery with a pretreatment MN technique in different ethnic/racial groups. Moving forward this may also inform how formulations can be tailored to individuals as we aim to achieve more personalized medicine.

### Subject demographics

A total of 111 subjects completed the study. According to self-identified ethnic/racial background, subjects were represented in the following 6 groups: Asian (n = 32), Black (n = 22), Latino (n = 23), White (n = 23), Native American (n = 1), and Bi-/multi-racial (n = 10). There was no significant difference in participants’ age between groups (*p* > 0.05). There were no significant differences in BMI between most groups, though the Black group had significantly higher BMI than the Asian group (*p* < 0.05). General demographics of the participants are described in Table 1.

**Table 1:** Demographics of subjects (n = 111).

Self-identified ethnic/racial background	# Of subjects	Age, years (range)	Gender	BMI, kg/m^2^
Asian	32	27.3 ± 8.9 (18–50)	M = 11; F = 21	23.3 ± 4.8
Bi-/multi-racial	10	20.6 ± 2.8 (18–27)	M = 2; F = 8	23.5 ± 1.0
Black	22	28.0 ± 7.0 (18–46)	M = 9; F = 13	28.0 ± 6.9
White	23	27.6 ± 8.2 (18–49)	M = 9; F = 14	26.0 ± 0.9
Latino	23	23.3 ± 6.2 (18–40)	M = 9; F = 14	27.2 ± 1.0
Native American	1	18	F = 1	19.9

Age and BMI are reported as mean ± SD.

### Baseline epidermal characteristics

#### Transepidermal water loss

TEWL measures the total amount of water lost through the skin under non-sweating conditions, and is generally used as a measure of the degree of skin barrier functionality^4,19^. In human subjects of different ethnic/racial background, results of TEWL have been contradictory^2,4,20^. In the current study, baseline TEWL measurements for each group were as follows (mean ± SD): 7.66 ± 1.66 g/m^2^ h for Asian, 7.93 ± 2.33 g/m^2^ h for Bi-/multi-racial, 5.25 ± 2.12 g/m^2^ h for Black; 6.71 ± 1.74 g/m^2^ h for White, 6.87 ± 1.87 g/m^2^ h for Latino, and 4.99 g/m^2^ h for Native American/Hawaiian. Baseline TEWL for the Black group was significantly lower than that of the Asian and Bi-/multi-racial groups (*p* < 0.05). These findings agree with results from Muizzuddin et al. which showed that African Americans have significantly lower TEWL values in comparison with East Asians^3^. In the current study there was no significant difference in baseline TEWL values between the Asian, Bi-/multi-racial, White, and Latino groups. While TEWL in White and Latino subjects was higher than that of Black subjects it was not significantly different, also in agreement with previous studies^20–22^.

#### Impedance spectroscopy

Impedance measures the response of a region of skin to an electrical current that is externally applied^23^. It has also been used as a reliable method to provide physiological information related to skin barrier function, describing such parameters as skin hydration, stratum corneum thickness, skin permeability, and condition of skin water channels^23,24^. Unlike TEWL measurements that can be very sensitive to the hydration status of the skin^25^, impedance can more readily measure small changes in electrical resistance of hydrated skin^18^. In the current study, mean (± SD) baseline impedance measurements for each group were as follows: 52.03 ± 16.34 kΩ for Asians, 50.46 ± 21.92 kΩ for Bi-/multi-racial, 77.64 ± 18.43 kΩ for Blacks, 60.05 ± 18.11 kΩ for Whites, 57.60 ± 16.83 kΩ for Latinos, and 64.5 kΩ for the Native American/Hawaiian subject. Baseline impedance of the Black group was significantly higher than the Asian, Bi-/multi-racial, White, and Latino groups (*p* < 0.05). While no study has compared baseline skin impedance in all the ethnic/racial groups investigated in this study, Bernstein et al*.* demonstrated a significantly higher baseline impedance in Black versus White subjects^26^, which is in agreement with our results. Likewise, Johnson and Corah showed that mean electrical resistance in Black skin was significantly higher than that in Whites^27^. In addition, high skin impedance can be related to high resistance to water diffusion through the skin, which may correspond with the lower TEWL values obtained in this study for Blacks^23^. The lower TEWL and significantly higher impedance in the Black subjects may also be attributed to the stronger skin barrier strength and stratum corneum cohesiveness, which has been previously reported for Black skin types^2,3^.

#### Skin color

The most obvious physical difference in skin of various ethnic/racial groups is the pigmentation and color^2^. Subjects in our study self-identified their race and ethnicity, though this does not provide a quantitative way to determine differences in pigmentation between groups. For this reason, we used a tristimulus colorimeter to quantify skin color. The CIELAB uniform color space (sometimes also referred to as CIE L*a*b*) is a well-accepted strategy to measure skin color. The CIELAB color space parameter L*, which describes the degree of lightness, is an appropriate technique to ascribe skin color to human subjects of different races^28^. Our results show a wide distribution of L* values within and between the self-identified ethnic/racial groups enrolled in our study (Fig. 3). The unitless L* values of subjects self-identifying as Black (40.61 ± 6.74) were significantly lower than those self-identifying as Asian (59.77 ± 7.62), Bi-/multi-racial (61.12 ± 5.79), White (64.67 ± 4.71), Latino (58.84 ± 7.21) and Native American/Hawaiian (60.37), *p* < 0.05. This agrees with other studies that also found L* values of self-identifying Black individuals to be significantly lower than those of self-identifying White, Asian, and Bi-/multi-racial subjects^29,30^.

#### Skin hydration

Skin hydration plays an important role in maintaining healthy skin, as adequate hydration helps to support optimum physical and mechanical skin properties^31,32^. In the current study, mean (± SD) unitless skin hydration measurements for each group were as follows: 38.32 ± 23.97 for Asians, 50.27 ± 21.60 for Bi-/multi-racial, 34.75 ± 18.49 for Blacks, 36.06 ± 18.81 for Whites, 35.61 ± 14.23 for Latinos, and 24.1 for the Native American/Hawaiian subject. While Bi-/multi-racial subjects overall had the highest baseline skin hydration, no significant differences in baseline hydration were observed between groups (*p* > 0.05). This is in accordance with previous work in which skin hydration was not significantly different between young women of African American, White, Chinese, and Mexican ethnic/racial backgrounds^33^.

### Microneedle treatments and micropore formation

MN treatment was well tolerated by all subjects, with no adverse reaction observed at the MN treatment sites. Notably we did not observe any hyperpigmentation at any MN treatment site, which is very important because darker skin tones have a higher risk of developing pigment changes^34^. One subject discontinued the study early due to a mild skin reaction to the waterproof medical tape used to secure the occlusive patches on the skin (this subject’s data was not included in the analysis). Immediately after MN application, TEWL and impedance measurements were repeated to ascertain the success of the MN application and formation of micropores. Under typical conditions of normal uncompromised skin, TEWL measurements are low at baseline and will increase in response to barrier disruption^15,35^. Impedance serves as a complementary technique to TEWL, as impedance for normal uncompromised skin is high and will decrease in response to barrier impairment^15,35^. Both techniques have been used to confirm micropore formation and study micropore closure time in human subjects^15,35,36^.

**Figure 1:**
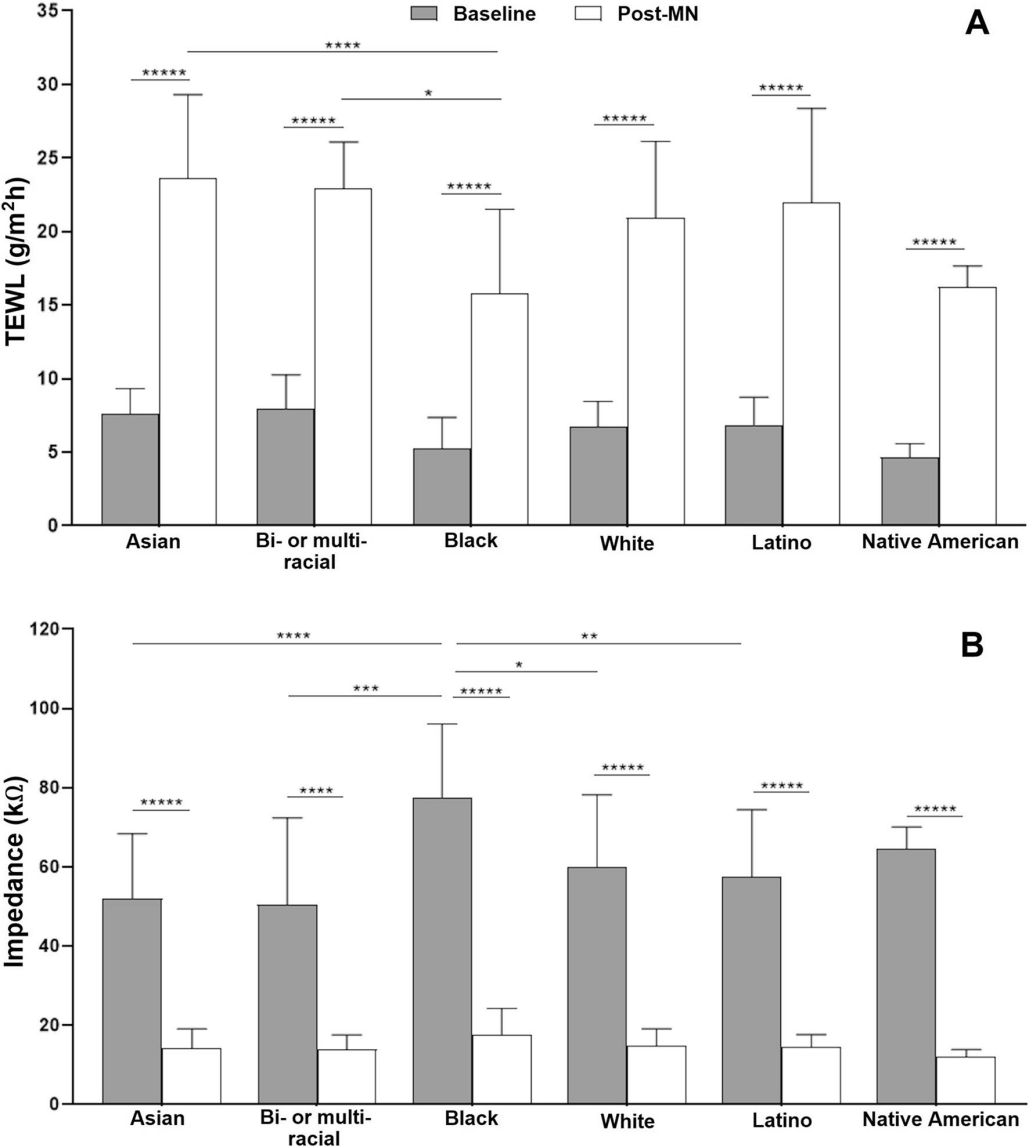
(**A**) Pre- and post-MN TEWL; (**B**) Pre- and post-MN impedance. Data presented as mean ± SD of the three MN sites. *p < 0.05, **p < 0.01, ***p < 0.005, ****p < 0.0001, *****p < 0.000001.

There was a significant increase in TEWL and decrease in impedance from baseline to post-MN application for the majority of MN-treated sites in all subjects, regardless of ethnic/racial group (*p* < 0.05, Fig. 1). These data suggest that MN application created a breach in the skin barrier, and therefore indirectly confirms the formation of micropores. There was no significant increase in TEWL values from baseline to post-MN application in two subjects, and they were not included in the TEWL analysis. However, these two subjects showed significant decrease in impedance from baseline to post-MN application, and thus they were included in the impedance analysis.

To compare changes in TEWL and impedance between groups, the mean of measurements at all three sites for each subject were used in the analysis. The mean (± SD) increase in TEWL from baseline to post-MN was 212.81 ± 58.83% in Asians, 203.12 ± 79.81% in Bi-/multi-racial, 240.95 ± 97.47% in Blacks, 228.25 ± 75.58% in Whites, 223.93 ± 82.31% in Latinos, and 200% in the Native American/Hawaiian subject. The corresponding decrease in impedance from baseline to post-MN was 69.28 ± 10.93% in Asians, 67.07 ± 13.27% in Bi-/multi-racial, 76.54 ± 7.76% in Blacks, 72.13 ± 11.0% in Whites, 70.51 ± 9.35% in Latinos and 80.1% in the Native American/Hawaiian subject.

The success of MN-assisted drug delivery, particularly that of solid MNs using the pretreatment strategy, depends on the formation of micropores in the skin. These micropores are essential for providing the pathway through which therapeutics can diffuse into the body and it is critical that they are formed effectively and reliably in individuals of all skin types. In the current study, it is interesting to note that while there were significant differences in baseline TEWL and impedance between some ethnic/racial groups, no significant differences in the baseline to post-MN change in TEWL and impedance were observed between groups. These data confirm that effective micropore formation occurred in all ethnic/racial groups represented in this study. This suggests that baseline TEWL or impedance of different skin types may not influence the outcome of a MN application procedure that is correctly performed, which is necessary for large-scale, global use of MNs for drug delivery.

### Micropore closure time

In using a pretreatment MN delivery technique, micropores are created in the skin and a formulation patch is applied over the MN-treated site for the drug to diffuse into the systemic circulation^18^. After successful creation of epidermal micropores, the time that the micropores remain open is the next critical parameter for drug delivery because of its direct effect on the length of time that drug will be absorbed through the skin. With an expected resealing of the micropores, the concept of micropore lifetime is particularly useful for defining the delivery window during which drugs can be transdermally absorbed to attain therapeutic plasma concentrations.

Micropore closure timeframe, however, may depend on innate skin barrier function characteristics that differ in people from different ethnic/racial backgrounds^15^. The uses of MN-assisted delivery are expanding rapidly in therapeutic areas ranging from the delivery of small drug compounds to large molecules such as vaccines and hormones. Some of the benefits of MN delivery are particularly well suited for use in developing countries, including reduction of biohazards from syringe/needle waste and minimizing the need for medical training for application^37^. There are also rapidly changing racial/ethnic demographic groups within the United States, and nearly all racial/ethnic groups could benefit from MN-assisted delivery^38^. All of these considerations imply that understanding how micropores behave after MN application in different skin types will be beneficial to developing successful MN delivery strategies and possibly tailoring transdermal delivery systems to specific patient groups.

In the present study, micropore closure time under occlusion was determined by converting skin impedance to admittance and comparing the admittance of MN-treated sites to occluded control sites at each timepoint using a Student’s *t*-test^15^. When there was no statistical difference in admittance between MN and control sites, it was determined that the micropores were closed (i.e., the micropores closed sometime in the 24 h since the previous measurement). Table 2 shows the timepoints at which micropores were determined to be closed in each group, based on the lack of statistical difference between MN and control sites.

**Table 2:** Micropore closure time determined by statistical analysis.

Ethnic/racial group	Time of micropore closure (number of subjects)	Mean time of micropore closure (h)
Asian (n = 32)	24 h (7) 48 h (23) 96 h (2)	44.1 ± 14.0^a,b^
Bi-/multi-racial (n = 10)	24 h (2) 48 h (6) 72 h (2)	48.0 ± 16.0^a^
Black (n = 22)	48 h (10) 72 h (7) 96 h (5)	66.5 ± 19.5^a^
White (n = 23)	24 h (2) 48 h (17) 72 h (4)	50.1 ± 12.4^a^
Latino (n = 23)	48 h (12) 72 h (8) 96 h (3)	61.1 ± 16.1^b^
Native American/Hawaiian (n = 1)	72 (1)	72

Data reported as mean ± SD.

^a,b^Differences in times of micropore closure were significantly different (*p* < 0.05) between groups sharing the same superscript letter.

The mean (± SD) time of micropore closure was significantly longer in Blacks (66.5 ± 19.5 h) when compared to Asian (44.1 ± 14.0 h), Bi-/multi-racial (48.0 ± 16.0 h), and White (50.1 ± 12.4 h) subjects; *p* < 0.05. Likewise, time to micropore closure was significantly longer in Latinos (61.1 ± 16.1 h) when compared to Asians; *p* < 0.05. No significant difference in micropore closure time was observed between the Black and Latino groups. There was no significant influence of age or BMI on micropore closure time within and between all studied ethnic/racial groups.

Studies that have investigated epidermal recovery in different ethnicity/races after acute epidermal perturbation reported faster recovery rate in Black subjects^20,39^. Our results did not follow the same trends as these previous observations, though there are some notable differences in methods that may explain the dissimilarities. Our results were obtained following barrier disruption with MNs, which is a different type of disruption than tape stripping that was used in previous work. The studies of tape stripped skin did not include occlusion of the skin sites after the barrier disruption, and in the current study the MN treated sites were occluded with patches between measurements. This can have a large influence on epidermal recovery; in fact, applying occlusive patches over MN treated skin is known to prolong micropore lifetime and safely permit longer drug delivery windows^13,36^.

#### Predicted micropore closure half-life

**Figure 2:**
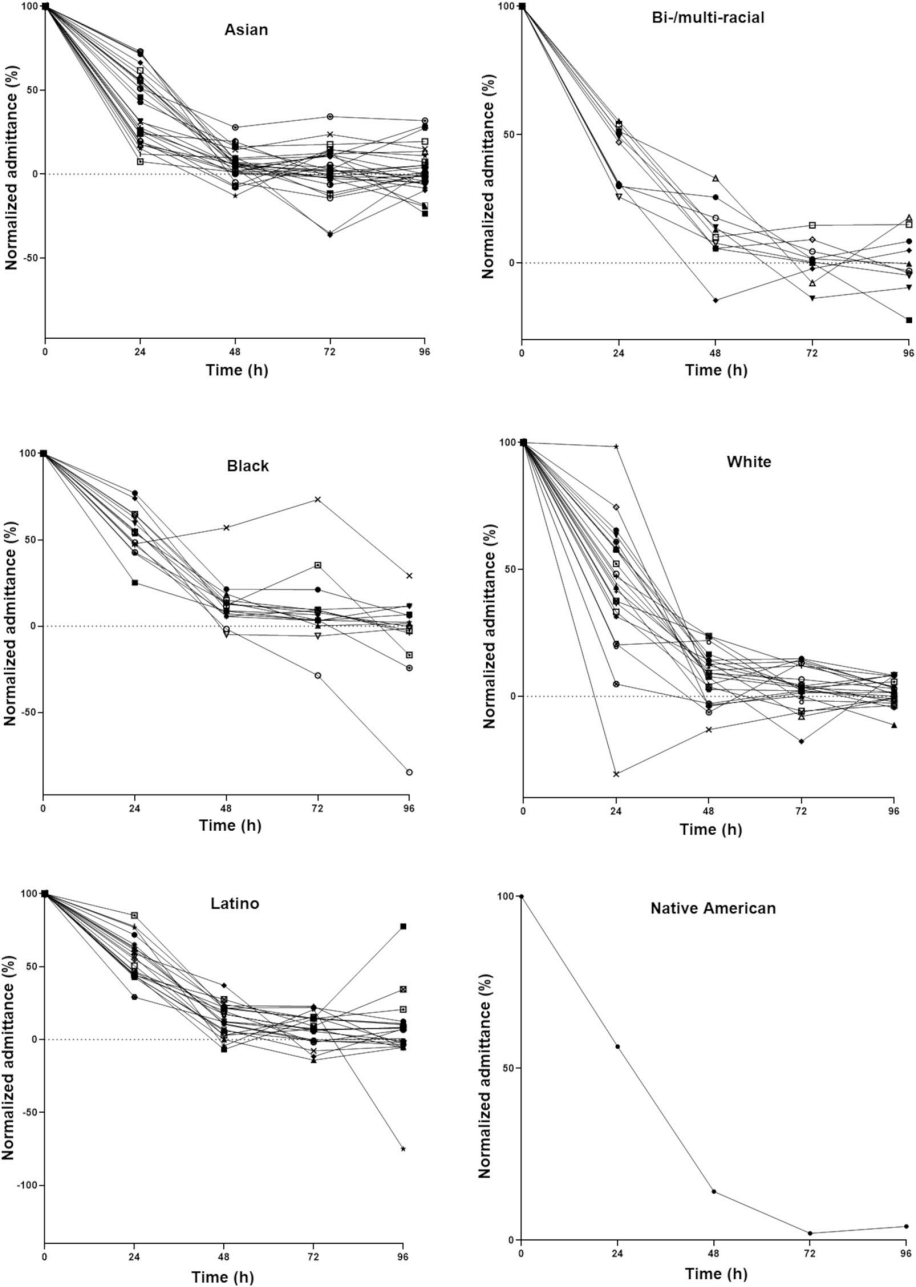
Representative normalized micropore admittance (%) of subjects within different ethnic/racial groups. Each line represents an individual subject; error bars are removed for graph clarity.

Micropore closure times were initially determined in this study in increments of 24 h simply based on statistical analysis, but in clinical settings it will be necessary to identify more specific timeframes that accurately demonstrate the MN-assisted drug delivery window. We devised a complementary method to estimate this timeframe in the form of a micropore closure half-life (t_1/2_). To estimate this, impedance values were transformed to admittance and normalized to the highest post-MN admittance in each subject. Brogden et al. logarithmically transformed normalized admittance values to determine t_1/2_ in an apparent first-order model^18^, though in the present study we fitted the logarithmically transformed data to a non-linear second-order polynomial model to accommodate for inter-subject variability in admittance observed in the different groups (Fig. 2). Generally the profiles considerably fit the model with R^2^ values > 0.9 (n = 66), > 0.8 (n = 22), > 0.7 (n = 9) and > 0.6 (n = 4), while ten profiles could not be fitted to the model.

The ranking of predicted t_1/2_ was as follows: Latinos, 20.4 ± 9.9 h > Blacks, 19.3 ± 9.0 h > Whites, 17.8 ± 11.0 h > Bi-/multi-racial, 17.4 ± 10.1 h > Asians, 14.1 ± 8.7 h > Native American/Hawaiian, 11.24 h. No significant differences in predicted t_1/2_ were observed between any of the studied ethnic/racial groups (*p* > 0.05). Based on general principles of pharmacokinetics, a window of 3–5 half-lives can be used to describe the timeframe at which ~ 87.5 to 97% of micropores are expected to be closed, thereby prohibiting further drug absorption through the micropores^15^. Based on this approach, the micropore closure window was calculated for each group: 61.2–102.0 h for Latinos, 57.9–96.5 h for Blacks, 53.3–88.8 h for Whites, 52.2–87.0 h for Bi-/multi-racial, 42.4–70.6 h for Asians, and 33.7–56.2 h for the Native American/Hawaiian.

Time to micropore closure determined from statistical analysis showed some agreement with the predicted closure window in some ethnic/racial groups (Table 3). The mean time to micropore closure for Asians was 44.1 ± 14.0 h, which was within the predicted micropore closure window of 42.4–70.6 h based on micropore t_1/2_. Likewise, mean micropore closure time in Blacks was 66.5 ± 19.5 h, which is also within the predicted closure window of 57.9–96.5 h. Predicted micropore closure windows for Bi-/multi-racial, Whites, and Latinos also overlapped with the mean micropore closure time derived from the statistical analysis. There was no overlapping of derived micropore closure time and predicted micropore closure window for the Native American/Hawaiian subject, though there was only one subject in this group. Overall, these results suggest that a model such as the one implemented here may be useful in predicting the timeframe over which micropores may be available for optimum drug delivery after MN application.

**Table 3:** Mean micropore closure time, predicted closure half-life, and closure window.

Ethnic/racial group	Micropore closure time (h)*	Predicted micropore closure half-life (h)	Predicted micropore closure window (h)
Asian	44.1 ± 14.0^a,b^	14.1 ± 8.7	42.4–70.6
Bi-/multi-racial	48.0 ± 16.0^a^	17.4 ± 10.1	52.2–87.0
Black	66.5 ± 19.5^a^	19.3 ± 9.0	57.9–96.5
White	50.2 ± 12.6^a^	17.8 ± 11.0	53.3–88.8
Latino	61.1 ± 16.1^b^	20.4 ± 9.9	61.2–102.0
Native American/Hawaiian	72	11.24	33.7–56.2

Data reported are mean ± SD; *Micropore closure time is estimated from *t*-tests comparing impedance at MN treated sites versus control sites at the same time point.

^a,b^Differences in times of micropore closure were significantly different (*p* < 0.05) between groups sharing the same superscript letter.

### Relationship of skin color and measured parameters

**Figure 3:**
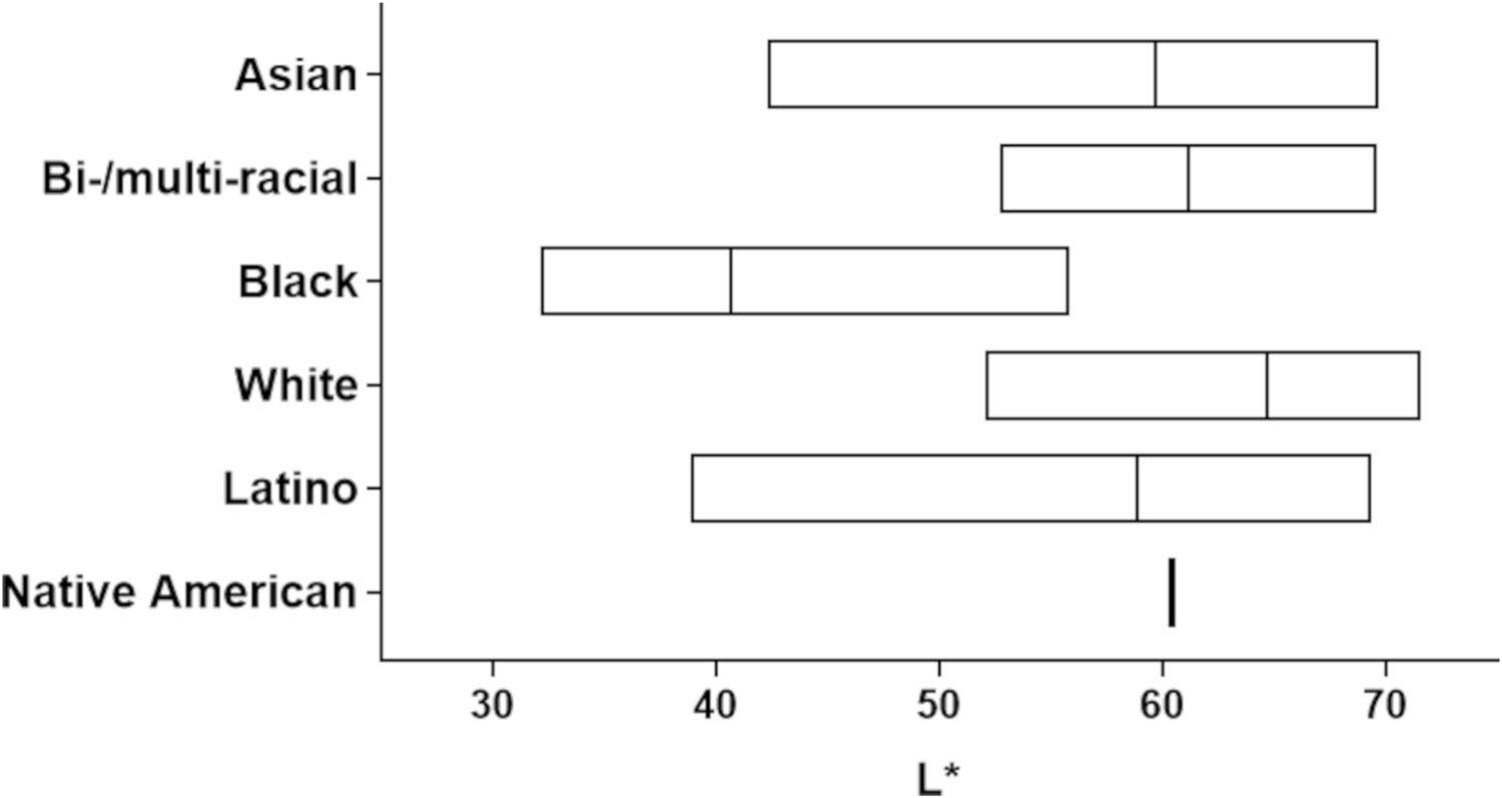
Color distribution among participants of self-identified diverse ethnic/racial groups showing (unitless) L* range. Boxes represent the range of values, and the line within each box represents the mean L*.

Studies that have investigated ethnic/racial differences in skin barrier often report self-identified ethnicity or skin type based on the Fitzpatrick classification scale^2,3,21,39^, though the Fitzpatrick classification system may have limitations in its use as an accurate predictor of self-reported race or appearance^40^. Other studies have demonstrated a close correlation between skin pigmentation and skin barrier functionality, with darker skin believed to have stronger skin barrier function^39,41,42^. Jeffrey et al. observed that skin pigmentation may have more significant influence on skin barrier functionality rather than self-declared race/ethnicity. They demonstrated that although baseline TEWL measurements did not vary based on race or skin type, it was evident in skin barrier function studies that darker skin pigmentation had better skin barrier function^39^. In our study we used reflectance spectroscopy as a quantitative technique for measuring skin color because it does not incorporate assumptions about the nature or amount of pigment in the skin^28,43^. The L* parameter of the tristimulus colorimetry system depicts lightness, ranging from black (0) to absolute white (100), and is the most sensitive of the trichromatic values to skin color change^43^. Considering the overlapping range of L* values between and within each self-reported ethnic/racial group in our study (Fig. 3), we chose to investigate correlations between subjects’ L* values, baseline skin barrier functionality parameters (TEWL, impedance, and hydration), and micropore closure time.

**Figure 4:**
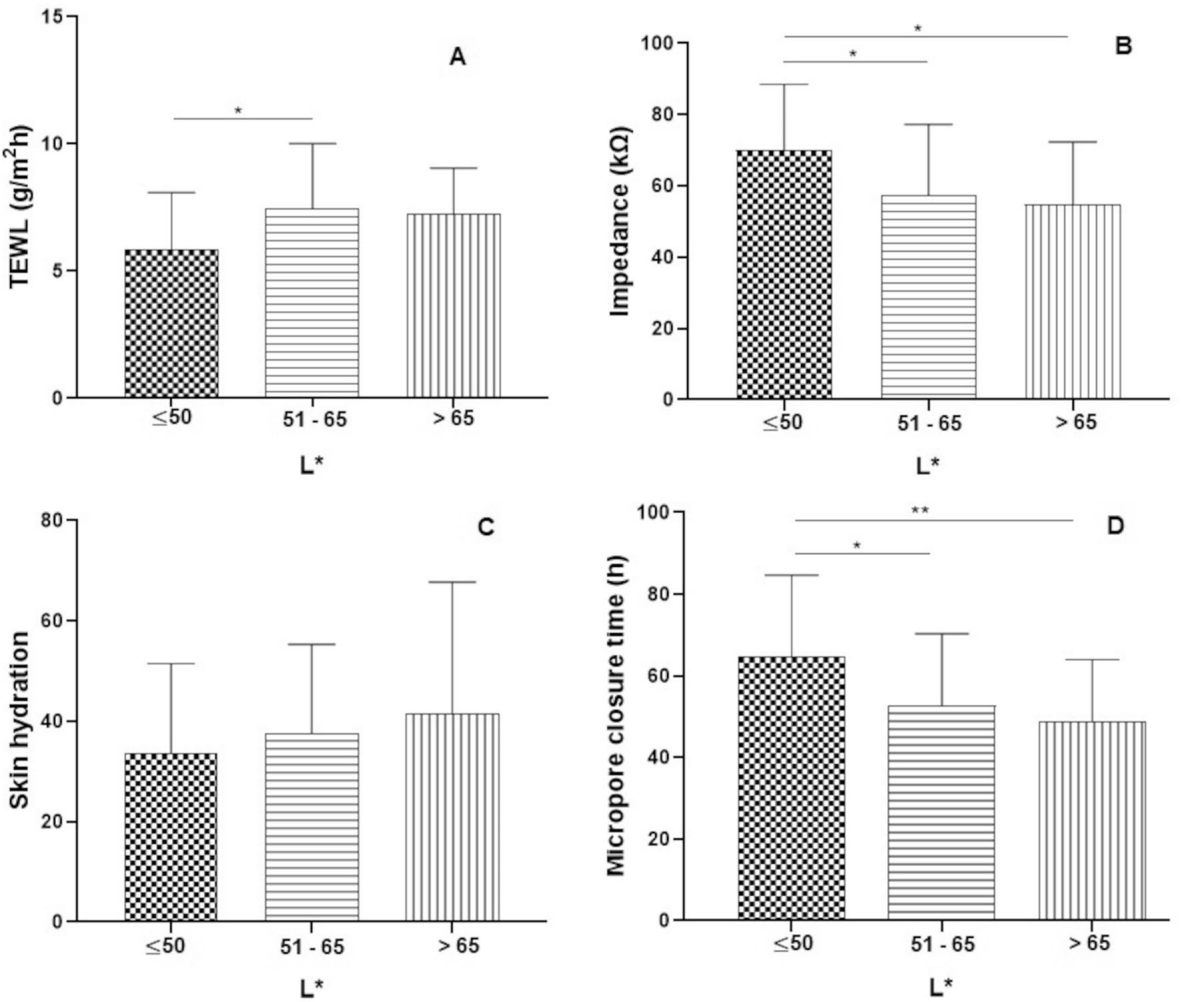
Representation of relationship between L* and baseline TEWL (**A**), baseline impedance (**B**), skin hydration (**C**), and observed micropore closure time (**D**). Bars represent mean ± SD. *p < 0.05, **p = 0.0055.

Studies have shown that darker skin such as that of Blacks have L* values approximately 50 and lower, while Whites have L* values considerably higher than 60^30,44^. In this study, to investigate the influence of skin color on measured parameters, we placed subjects into three categories based on unitless L* values: ≤ 50, 51–65, and ≥ 65 to accommodate subjects with considerably low (darker), medium, and high (lighter) L* values irrespective of their self-reported ethnic/racial background. Ethnic/racial distribution within each L* category is shown in Table 4 along with the micropore closure time, predicted micropore closure t_1/2_, and predicted micropore closure window when data are re-categorized based on L* instead of self-identified race/ethnicity. Figure 4 shows the relationship between L* value categories and TEWL, impedance, skin hydration, and micropore closure time.

**Table 4:** Subject self-identified ethnicity/race and categorization of L* values.

L* Category	Self-identified ethnic/racial group	Micropore closure time (h)	Predicted micropore closure t_1/2_ (h)	Predicted micropore closure window (h)
< 50 (n = 26)	Asian (n = 3)	64.6 ± 20.1	18.0 ± 9.0	47.8–79.6
	Black (n = 20)			
	Latino (n = 3)			
51–65 (n = 60)	Asian (n = 21)	52.8 ± 17.6	16.7 ± 9.1	42.6–71.0
	Bi-/multi-racial (n = 7)			
	Black (n = 2)			
	White (n = 11)			
	Latino (n = 18)			
	Native American/Hawaiian (n = 1)			
> 65 (n = 25)	Asian (n = 8)	49.0 ± 14.7	17.7 ± 7.2	44.7–74.5
	Bi-/multi-racial (n = 3)			
	White (n = 12)			
	Latino (n = 2)

#### TEWL and L*

Baseline TEWL values were significantly different only between the groups with L* values ≤ 50 versus 51–65 (*p* < 0.05). Both of these groups contained subjects identifying as Black, Asian, and Latino, and the 51–65 L* group also contained subjects self-identified as Bi-/multi-racial, White and Native American/Hawaiian. It is notable that comparisons between Blacks and Latinos showed no significant difference in TEWL values when subjects self-identified as any of the ethnic/racial groups; however, when L* categorization was used, there were significant differences in TEWL even though the L* categories had subjects from the self-identified Black and Latino ethnic/racial groups (Fig. 4). This suggests that objective measurements of L* for categorizing skin color may give better information on differences in TEWL values between individuals rather than self-reported ethnic/racial makeup. This observation may be supported by the study of Jeffrey et al. that found no significant difference in barrier function between African Americans and darkly pigmented Latinos and Filipinos, suggesting that skin color parameter may be a more relevant determinant of barrier function than race^39^.

#### Impedance and L*

Figure 4B shows the relationship between L* values and skin impedance. Skin impedance in subjects with L* values ≤ 50 was significantly higher (*p* < 0.05) than those with higher L* values (51–65 and > 65). While no study has directly investigated the relationship of skin color and electrical resistance, there are previous reports showing that Blacks exhibit higher skin electrical resistance and our results correlate with these findings^27^. In our study, baseline impedance was significantly higher in Black subjects when data were grouped based on self-identified ethnicity. Baseline impedance correlated with L* values followed the same trend, showing higher impedance in subjects with lower L*. One variable that may contribute to this effect is the hydration state of the skin. Lower electrical conduction (higher resistance) has been reported in dry skin vs. wet skin^24,45^, and darker skin types have decreased hydration due to low ceramide content^4^. Although no significant difference in skin hydration was observed based on self-identified ethnic/racial groups or L* values, subjects with L* values ≤ 50 demonstrated overall lower skin hydration compared to subjects with higher L* values. This signifies a dryer stratum corneum and may contribute to resultant higher skin impedance.

#### Relationship of L* and micropore closure

When analyzing the data according to self-identified ethnicity/race, mean micropore closure time was significantly longer in Blacks versus Asians, Bi-/multi-racial, and Whites, and significantly longer in Latinos versus Asians. With L* groupings, mean micropore closure time in subjects with L* ≤ 50 (64.6 ± 20.1 h) was significantly longer than those with L* of 51–65 (52.8 ± 17.6 h) and > 65 (49.0 ± 14.7 h), *p* < 0.05. This suggests that subjects with darker skin color exhibited longer average micropore closure time than lighter skin, even though these L* groupings are composed of overlapping self-identified ethnic/racial groups as shown in Table 4. Thus, skin type in terms of color may be a better parameter to evaluate micropore closure time after MN application. The predicted micropore closure half-life of subjects with L* ≤ 50 (18.0 ± 9.0 h) was longer than those of subjects with L* values of 51–65 (16.7 ± 9.1 h) and L* values > 65 (17.7 ± 7.2 h), though interestingly these differences were not statistically significant. In like manner, predicted micropore closure windows were 47.8–79.6 h for subjects with L* ≤ 50, 42.6–71.0 h for L* of 51–65, and 44.7–74.5 h for L* > 65.

## Limitations

There are some limitations to this work. Our subject pool included only a single Native American/Hawaiian subject, and while these data should be interpreted with high level of caution we felt it was important to include these data due to the limited literature available studying MN use in diverse skin types. Several investigators were involved in conducting the study, and inter-investigator differences in MN application could contribute to some variability in the data. An applicator does not exist for the MN arrays used in this study, but we actively tried to mitigate this potential limitation: the same application procedures were followed by all investigators, and applications at all three MN sites on a subject were performed by the same investigator to eliminate inter-site variability. Micropore lifetime windows were calculated based on surrogate measurement techniques (TEWL, impedance) and we did not correlate the findings with pharmacokinetic studies demonstrating drug permeation through the skin. However, TEWL and impedance measurements are known to correlate closely with pharmacokinetic data in MN studies^13,14^. Our calculations in the White group closely match data from these previous studies, so we do not expect the use of surrogate measurements to be a significant weakness. In this study we presented and compared data regarding basic skin parameters such as TEWL, hydration, impedance, and color. In future studies it will be important to further explore how skin properties such as elasticity and thickness can influence the outcome of MN application, and how the use of imaging techniques such as optical coherence tomography (OCT) may be valuable in understanding MN effects on the skin.

## Conclusion

We have demonstrated for the first time that differences exist in micropore closure time after MN application in a group of subjects with differing epidermal properties and varied racial/ethnic backgrounds. Objectively measured skin color, rather than ethnic/racial self-identification, may be a better parameter to investigate and explain differences in skin response to MN application, as darker skin tends to exhibit lower TEWL, higher impedance, and longer micropore closure time irrespective of self-identified ethnicity/race. These findings have important implications for safety and efficacy when using MNs to transdermally deliver drugs in diverse patient populations because of the possible differences that may arise in drug delivery windows after MN application. Next steps for this research will include drug delivery studies that can correlate micropore closure time with drug permeation in different ethnic/racial groups treated with MNs.

## Materials and methods

### Microneedle arrays and occlusive coverings

**Figure 5:**
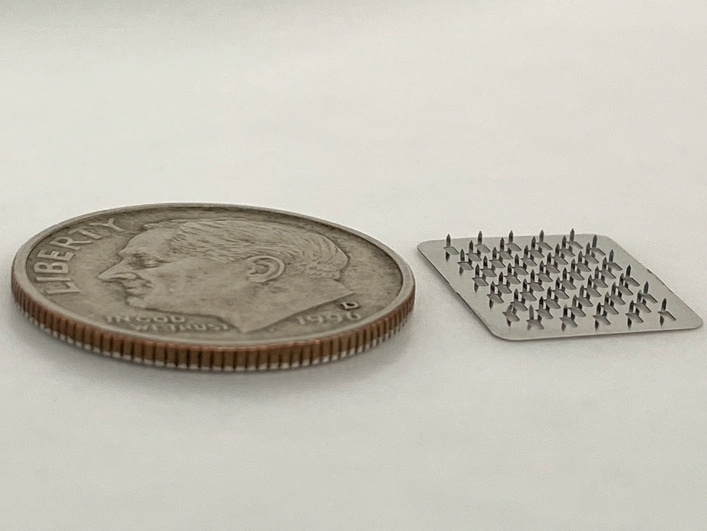
A stainless steel MN array used in the study, next to a US dime for size comparison.

MNs used in these studies were stainless steel arrays containing 50 MNs arranged in a 5 × 10 configuration (Tech Etch, Plymouth, MA). The MNs were 800 µm in length and 200 µm in width at the base (Fig. 5). Each array was assembled into a patch with AR7717 adhesive backing (Adhesives Research, Glen Rock, PA) to hold the array securely in contact with the skin during application. The adhesive backing compensates for the mechanical structural difference between the flexible skin tissue and the rigid microneedles^35^. All assembled micropatches were individually sterilized before use.

Small occlusive coverings/patches used to cover the MN treatment sites were fabricated in-house by affixing a silicone rubber ring (FDA White Buna 60 durometer, Ilene Industries Inc, Shelbyville, TN) to a drug impermeable membrane backing (Scotchpak 1109 SPAK 1.34 MIL heat-sealable polyester film; 3 M, St. Paul, MN) with the aid of double-sided tape (3 M, Saint Paul, MN).

### Enrollment of research subjects

All study procedures were approved by the University of Iowa Institutional Review Board and followed the guidelines adopted by the World Medical Association Declaration of Helsinki. Study activities were performed in the Clinical Research Unit at the University of Iowa Hospitals and Clinics. Written informed consent was obtained from all subjects prior to study enrollment. Healthy volunteers between the ages of 18–50 years with no history of dermatologic disorders were recruited and interviewed to determine eligibility for enrollment. Subjects were excluded for the following reasons: use of daily medications (oral contrceptives and vitamins were allowed), medical conditions affecting the immune system, local skin conditions at the MN application sites, or if pregnant/nursing. Other exclusion criteria included inability to give consent, previous adverse reaction to MN treatment, and known allergy to latex, rubber, aloe, or other adhesive tapes.

### Measurement of epidermal properties

Hydration measurements were made with a Corneometer probe (CM 825, Courage + Khazaka electronic, Köln, Germary) connected to a multi probe adapter system (MPA 6, Courage + Khazaka electronic, Köln, Germary). The probe was gently placed on the skin site for approximately 5 s until the hydration value was recorded in arbitrary units on the MPA CTplus software (Courage + Khazaka electronic, Köln, Germary). Skin color was measured using a tristimulus colorimeter (ChromaMeter CR-400, Konica Minolta, Japan). The colorimeter was calibrated daily against a white plate provided by Konica Minolta. Measurements were made by placing the instrument head gently on the skin site for approximately 5 s while the color reflectance was measured. The L* values representing the light–dark axis were recorded in arbitrary units. Transepidermal water loss (TEWL) measurements were made using an open chamber evaporimeter (cyberDERM Inc, Broomall, PA). The evaporimeter was placed on the skin surface for approximately 30–60 s or until the reading stabilized. TEWL was measured in untis of g/m^2^ h, recorded by the software. Impedance measurements were made using an experimental setup similar to methods described in previous studies^15,36^. Ag/AgCl wet gel foam measurement electrodes (Series 800 electrodes; S&W Healthcare Corporation, Brooksville, FL) and a reference electrode (Superior Silver Electrode with PermaGel; Tyco Healthcare Uni-Patch, Wabasha, MN) were connected to an impedance meter (EIM 105-10 Hz Prep-Check Electrode Impedance Meter; General Devices, Ridgefield, NJ) using lead wires. The meter applied a low frequency (10 Hz), alternating current modified with a 200 kΩ resistor in parallel (IET labs, Inc., Westbury, NY). Impedance measurements were made in triplicate at each site.

### Microneedle insertion

The microneedle application protocol followed previously published procedures^15,18,35^. At the MN treatment sites, the skin was cleaned with a sterile alcohol wipe and allowed to dry before MN application. Following baseline epidermal measurements, the MNs were inserted by pressing the micropatch (MN array on an adhesive backing) against the skin site for approximately 15 s using gentle thumb pressure. The micropatch was removed from the skin, rotated 45º, and reapplied for another 15 s to create a total of 100 micropores at each MN treatment site. The micropatches were discarded immediately after use and a new micropatch was used for each site. Following MN insertion, the skin was covered with a sterile occlusive covering that was made in-house (described above).

### Clinical study protocol

**Figure 6:**
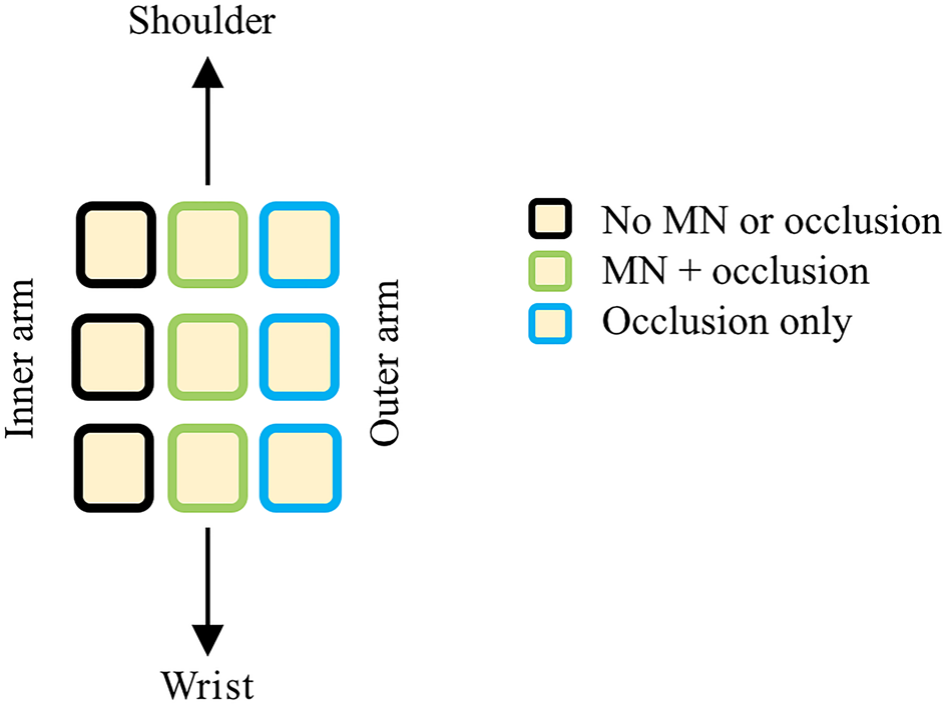
Schematic of the placement of all 9 sites on the upper arm.

Subjects’ age, gender, height, weight, and self-identified ethnicity/racial group were recorded at enrollment. At each visit, subjects sat quietly in the clinic roon for 30 min to acclimate to room temperature (~ 25 °C) before any study procedures were performed. Nine sites on the upper arm were identified and outlined with a marker, and the location and configuration of sites were the same for all subjects (Fig. 6 shows a schematic of how the patches were arranged). The upper arm was selected for the measurement sites because it represents an anatomical location that would be reasonable for patch wear in a clinical treatment setting. The sites received the following treatments: MN treatment + occlusion (n = 3 sites), occlusion only (no MN treatment) (n = 3 sites), and no MN or occlusion (n = 3). The occlusive patches were covered with 3M Tegaderm transparent medical tape (3M, St. Paul, MN) to allow the patches to stay in place and to protect the sites from water.

Baseline hydration, skin color, TEWL, and impedance were measured at all sites. Following MN treatment, TEWL and impedance measurements were immediately repeated. After occlusive patches were applied to the relevant sites, subjects left the clinic for the day and then returned to the Clinical Research Unit every 24 h for 4 days following MN application. Upon return each day, subjects sat in the procedure room for 30 min to acclimate to local conditions. Occlusive coverings were removed and excess moisture on the skin surface was gently blotted with soft paper wipes. Hydration, colorimetry, and impedance measurements were made at all sites and fresh patches were applied each day (TEWL measurements were only collected after the initial MN application on day 1). In order to minimize the time that the skin was exposed to the air, after an occlusive patch had been removed all measurements were made at that site and fresh occlusive coverings were replaced before moving on to the next site.

## Data analysis

### Formation of epidermal micropores

Mean TEWL and impedance values for each individual MN site were used to calculate the % increase in TEWL and % decrease in impedance from pre- to post-MN application. Micropore formation was confirmed when there was a significant difference between pre- versus post-MN measurements of TEWL and impedance. Any sites that did not have a statistically significant difference, suggesting inadequate micropore formation, were not included in the analysis.

### Calculation of micropore closure time

Impedance measurements were used to determine the micropore closure time. The mean of three impedance measurements was calculated for each site pre- and post-MN treatment and on all subsequent study days. Individual measurements that differed by ≥ 125% from other measurements at the same site were excluded from the analysis as outliers, similar to previous studies^15,35^. Three independent, parallel pathways for electrical current are present with this impedance setup: resistor box (Z_box_), intact skin (Z_skin_, pre-MN baseline), and micropores (Z_pores_). The impedance measurements give a value for Z_total_ that is comprised of the three pathways. Because Z_total_, Z_box_, and Z_skin_ are known, micropore impedance can be calculated according to Eq. 1^15,35^:

1 $$Z_{total} = \frac{1}{{\frac{1}{{Z_{box} }} + \frac{1}{{Z_{skin} }} + \frac{1}{{Z_{pores} }}}}$$

Z_skin_ was estimated from the occluded control sites on all days except baseline. This approach was used because these control sites were under the same occluded conditions as the MN-treated sites, which better accounts for the effect that occlusion and hydration may have had on the measurements.

Impedance measurements were converted to admittance, *Y*. Admittance is inversely proportional to impedance, and transforming the data in this way provides two distinct advantages. First, admittance values follow the same trend as TEWL (i.e., intact skin baseline values are low and increase significantly when the barrier is compromised). Since TEWL is the most widely recognized method for assessing barrier function, this makes the data overall easier to interpret. Second, transforming the data in this manner allows general pharmacokinetics equations to be used in the data analysis, thus allowing calculation of a more precise micropore closure half-life.

Micropore closure time was first simplistically determined by comparing the admittance of MN-treated sites to occluded untreated (non-MN) sites at each time point using Student’s *t*-tests. The micropores were determined to be closed at a given timepoint when there was no statistically significant difference between the admittance of the MN-treated sites and occluded non-MN sites on the same day. Each subject served as their own control.

### Micropore closure kinetics modeling

While a simple statistical comparison between MN-treament sites and control sites gives a general idea of the time by which the microproes are closed, it does not allow more precise calculations of a predicted micropore closure half-life. In order to determine these, admittance was normalized to the highest post-MN admittance and contributions from the occluded control site were subtracted to remove any effect that may be attributed to hydration status of the site. Based on previous studies^15,35^, normalized admittance values were logarithmically transformed and plotted against time (h). The data were fitted to a second-order polynomial using Microsoft Excel software, from which half-life of micropore closure was derived for each subject according to Eq. (2) (*a* and *b* are model derived parameters):

2 $${\text{t}}_{{{1}/{2}}} = \frac{{ - b \pm \sqrt {b^{2} - 4a\ln 2} }}{2a}$$

In order to prevent over fitting the model by having an R^2^ value of 1, at least four time points (0, 24, 48, 72 h) for each subject were used to fit the second-order polynomial model. Subjects (n = 15) whose admittance values at the MN-treated sites were lower than the occluded untreated control sites on or before 48 h (signifying that the micropores were closed by that timepoint) were excluded from the model. Subjects whose admittance values at the occluded untreated control sites were higher than those of the MN-treated sites only at 72 h were included in the model, and in such scenario, the logarithimically transformed admittance values were equated to zero.

### Statistical analysis

Student’s *t*-tests were performed to compare impedance and TEWL measurements pre- and post-MN treatment at each site, and to statistically estimate micropore closure time by comparison between control vs. MN-treated site. Influence of age, BMI, and self-identified ethincity/race on measured parameters were compared using one-way ANOVA. In addition, the influence of skin color (recorded as the L* value from the colorimeter) on measured parameters was also compared using one-way ANOVA. For all analyses, *p* < 0.05 was considered statistically significant. GraphPad Prism Software (GraphPad Software, San Diego, CA) was used for all statistical analyses.
